# A Severe Case of Spontaneous Intracranial Hypotension in an Adult Asian Male Improved With Trendelenburg Positioning: A Case Report

**DOI:** 10.7759/cureus.60199

**Published:** 2024-05-13

**Authors:** Nathan Enrico T Pineda, Cybele Liana D Go, Ma. Cristina M Valdez

**Affiliations:** 1 Institute for Neurosciences, St. Luke's Medical Center, Quezon City, PHL

**Keywords:** brain herniation, spontaneous cerebrospinal fluid leak, trendelenburg, bilateral subdural hematoma, spontaneous intracranial hypotension

## Abstract

Non-traumatic bilateral acute subdural hematomas are a rare occurrence. Etiologies typically include, but are not limited to, cortical artery bleeding, vascular lesions, coagulopathies, and spontaneous intracranial hypotension. We report a case of a 45-year-old Korean male with no known co-morbid illnesses nor history of head or neck trauma, who came to the emergency department due to a 10-day history of dizziness and head heaviness, followed by disorientation and drowsiness. The patient was diagnosed with bilateral acute subdural hematoma; hence, a bilateral posterior parietal craniotomy with evacuation of hematoma was performed. Neurologic status initially improved remarkably; however, during rehabilitation, there was recurrence of acute bilateral subdural hematoma requiring repeat surgical evacuation. There was no clinical improvement after the repeat surgery, and his condition further deteriorated in the neurosciences critical care unit showing signs of rostrocaudal deterioration at the level of diencephalon. A plain cranial CT scan was performed, which showed central herniation and “brain sagging.” A diagnosis of spontaneous intracranial hypotension was considered; thus, the patient was managed by positioning him in the Trendelenburg position alternating with flat position on the bed. A search for the cerebrospinal fluid leak was commenced by performing a whole spine MRI constructive interference in steady state (CISS) protocol, which showed a longitudinal spinal anterior epidural cerebrospinal fluid leak from spinal level C2 to T1. Radionuclide cisternography did not provide definitive scintigraphic evidence of a leak. The patient gradually improved and was eventually transferred out of the neurosciences critical care unit. After days of rehabilitation in the hospital, the patient was discharged ambulatory with minimal support.

## Introduction

Subdural hemorrhage in adults typically arises from traumatic head injury, which constitutes the predominant etiology [1]. Conversely, non-traumatic subdural hematomas are rare occurrences, which can be caused by ruptured aneurysms or arteriovenous malformations, intracranial tumor bleeding, and coagulopathy. Moreover, spontaneous intracranial hypotension due to cerebrospinal fluid leak is an infrequent but possible cause of subdural hemorrhage [2,3]. We report a case of an adult male with no history of headache nor trauma who presented with bilateral subdural hematoma, which recurred despite surgical drainage. Spontaneous intracranial hypotension was suspected and was confirmed by cranial MRI, with subsequent whole spine MRI revealing a cerebrospinal fluid leak in the upper cervical spine. Effective management ensued through conservative therapy.

## Case presentation

**Figure 1:**
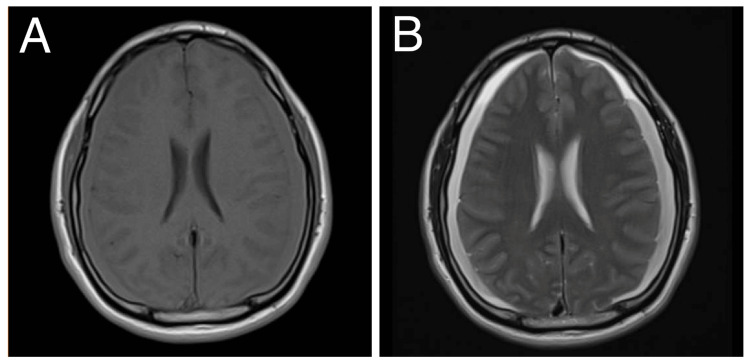
Non-contrast cranial MRI in (A) T1 and (B) T2 axial views showing a hyperacute to acute subdural hemorrhage along both cerebral convexities.

A 45-year-old male with no known comorbid illnesses presented at the emergency room with a 10-day history of non-rotatory dizziness, which was not associated with headache, vomiting, blurring of vision, extremity weakness or numbness, gait imbalance, fever, convulsions, or loss of consciousness. There was no history of trauma. Upon arrival at the emergency department, the patient was drowsy but not in cardiorespiratory distress. He had elevated blood pressure at 150/90 mmHg. The Glasgow coma scale (GCS) was 12 (E3V4M5), with unremarkable cranial nerve examination. Motor examination showed grade 4 muscle strength on manual muscle testing in all extremities. The reflexes were normal, and there was no nuchal rigidity. A non-contrast cranial MRI showed hyperacute to acute subdural hemorrhage along both cerebral convexities (Figure 1).

Hematologic workup showed erythrocytosis and homocysteinemia, while other bleeding parameters were within normal limits (Table 1).

**Table 1:** Pertinent laboratory results with reference interval PT, prothrombin time; PTT, partial thromboplastin time; INR, international normalized ratio; SGOT, serum glutamic oxaloacetic transaminase; SGPT, serum glutamate pyruvate transaminase

Test	Result	Reference Interval
Hemoglobin	17.6 g/dL	13.0-17.0 g/dL
Hematocrit	49.00%	40-52%
Platelet count	286,000/mm^3^	150,000-400,000/mm^3^
PT	11.4 seconds	11.9-14.2 seconds
PTT	27.2 seconds	29.5-39.9 seconds
INR	1.01	0.90-1.19
SGOT	14 U/L	0-34 U/L
SGPT	27 U/L	10-49 U/L
Homocysteine	26.49 umol/L	3.70-13.90 umol/L
Factor V Leiden	1.1	>0.8
Protein C	99%	70-140
Protein S	86%	60-140
Antithrombin III	103%	80-120%
Fibrinogen	4.0 g/L	2.0-4.0 g/L
Thrombin time	15.5 seconds	14-21 seconds
Bleeding time	9 minutes 30 seconds	2-10 minutes
Clotting time	7 minutes	5-15 minutes

**Figure 2:**
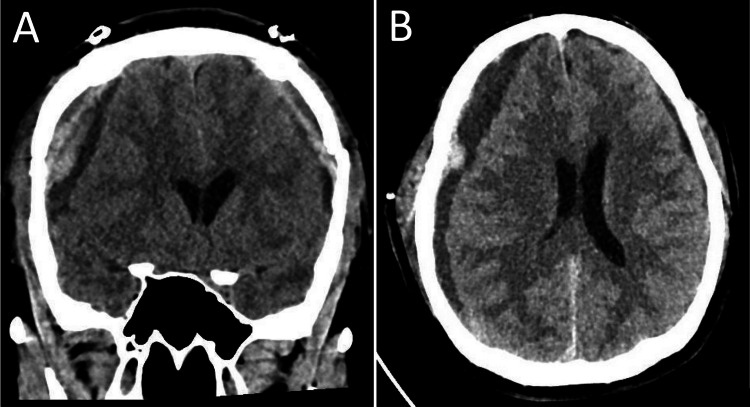
Plain cranial CT scan in (A) coronal and (B) axial views showing recurrence of subdural hematoma with leftward midline shift.

The patient was managed with medical decompression using mannitol at 1g/kg given as a bolus infusion followed by 0.5 g/kg every six hours. Despite medical decompression, his sensorium continued to deteriorate with a GCS of 7 (E1V1M5); hence, a bilateral craniotomy and evacuation of hematoma were performed. Postoperatively, the patient’s condition improved with no focal neurologic deficits, and he was transferred to the ward. On the fourth postoperative day, the patient had episodes of postural dizziness and doubling of vision during his physical therapy sessions. There were no complaints of headache, decrease in sensorium, or vomiting. The neurologic examination did not show any nystagmus or extraocular motor palsy. On the eighth postoperative day, he was noted by his wife to have prolonged periods of drowsiness during physical therapy and would prefer to stay in bed the entire day. On the 12th postoperative day, wax and waning sensorium persisted with a transient episode of confusion during conversation, accompanied by urinary incontinence while at rest. A two-hour video electroencephalogram (EEG) was subsequently performed, which showed no evidence of electrographic or clinical seizures during the monitoring. A series of plain cranial CT scans did not show recurrence of the bilateral subdural hematoma. Thyroid function test showed decreased thyroid-stimulating hormone (TSH) of 0.05 uIU/mL, with normal free T3 (FT3) and free T4 (FT4) at 2.56 pg/dL and 1.13 ng/dL, respectively. The rest of the metabolic evaluations were unremarkable. He was referred to an endocrinologist and was diagnosed with sick euthyroid syndrome. On the 14th hospital day, the patient’s condition deteriorated, and he became stuporous with anisocoria (right pupil was 3 mm, non-reactive; left pupil was 2 mm, briskly reactive). A repeat plain cranial CT scan showed recurrence of bilateral subdural hematoma, more prominent on the right cerebral convexity, with significant leftward midline shift (Figure 2).

The patient underwent bilateral frontal mini-craniotomy with endoscopy-assisted evacuation of hematoma, with no intraoperative complications, and was transferred to the neurosciences critical care unit for postoperative monitoring, with no improvement in neurologic status. Medical decompression with hypertonic saline was continued.

On the first postoperative day, a repeat plain cranial CT scan revealed no recurrence of bilateral subdural hematoma. Despite improved cranial CT scan results, the persistence of decreased sensorium and progression of anisocoria (right pupil measuring 5 mm, non-reactive; left pupil measuring 3.5 mm, reactive) prompted further workup. A non-contrast cranial MRI was performed to rule out an acute infarction in the midbrain (top of the basilar syndrome); however, it did not show any acute infarcts or parenchymal hemorrhage. A possibility of non-convulsive status epilepticus was likewise considered; hence, a two-hour video electroencephalogram (EEG) was performed, which showed generalized intermittent delta-theta slowing of the background activity. The patient was then referred to a vascular neurosurgeon for cerebral catheter angiography with transarterial embolization of the right middle meningeal artery. Intraoperative examination did not reveal any arteriovenous malformation, aneurysm, or fistulas. The patient remained obtunded and anisocoric with decerebrate posturing.

**Figure 3:**
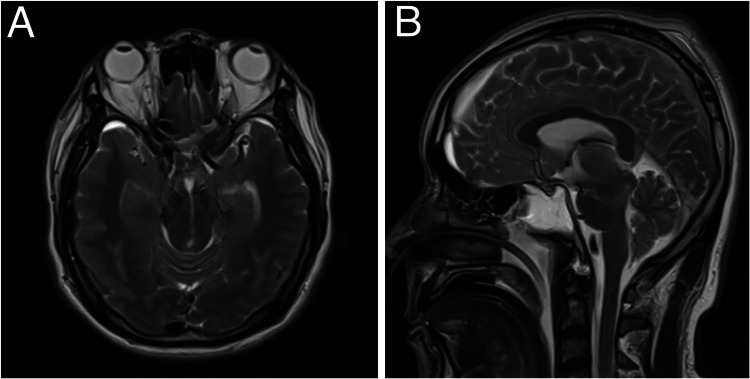
Non-contrast cranial MRI in (A) axial T2 and (B) sagittal T2 sequences showing compression of the bilateral midbrain and obliteration of the perimesencephalic sulcus.

**Figure 4:**
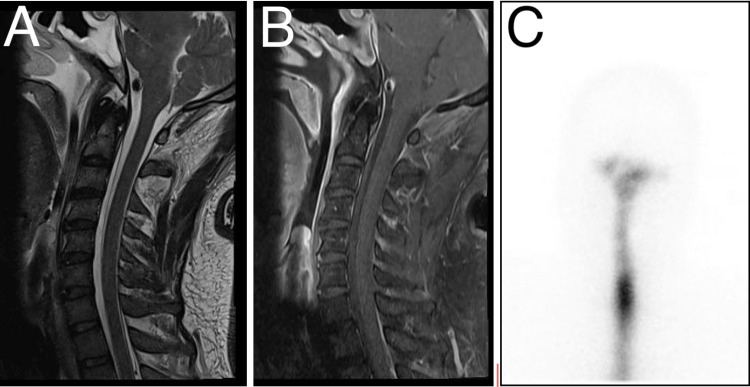
Cervical MRI CISS protocol in (A) T2 and (B) contrast sagittal views showing a longitudinal CSF collection in the anterior extradural space from the level of C2 down to the level of T1, effacing the thecal sac. (C) Radionuclide cisternography showing no diffusion of radioisotope in the extra-arachnoidal space. CISS, constructive interference in steady state; CSF, cerebrospinal fluid

Review of the neuroimaging showed progressing central herniation with signs of brain sagging (Figure 3); hence, a clinical impression of spontaneous intracranial hypotension was strongly considered. The patient was then placed on Trendelenburg positioning for one and a half hours, with the body lying flat on the back on a 30-degree incline with the feet elevated above the head, alternating with two and a half hours with the body lying flat on the bed, with the head elevated at 30 degrees to facilitate enteral feeding and prevent aspiration. This was done in six intervals per day. To further search for the cerebrospinal fluid leak, a whole spine MRI with contrast was requested. A constructive interference in steady state (CISS) protocol was added to provide better spatial resolution and cerebrospinal fluid-to-soft tissue delineation. Using this protocol, a longitudinal spinal anterior epidural cerebrospinal fluid leak from spinal level C2 to T1 was demonstrated (Figures 4A, 4B). The rest of the study showed thoracic and lumbar degenerative spondylosis.

Surgical repair was contemplated at this point, provided that the exact location of the leak must be established preoperatively. This prompted a radionuclide cisternography, which did not provide definitive scintigraphic evidence of a leak (Figure 4C); hence, no operative management was done.

After five days of conservative management with the previously described alternate positioning, the patient’s condition progressively improved. He eventually regained full consciousness after 11 days and was subsequently extubated. A non-contrast cervical spine MRI was obtained (10 days from the initial whole spine MRI), which showed a stable extent of extradural cerebrospinal fluid leak, suggesting closure or resolution of the leak site. The patient was transferred out of the neurosciences critical care unit with continuation of conservative treatment with bed rest, hydration, and rehabilitation. Since then, the patient has had no recurrence of dizziness, double vision, and decrease in sensorium. The patient was discharged ambulatory with minimal support and no recurrence of symptoms at follow-up. He was able to fly back to Korea after one month.

## Discussion

Spontaneous intracranial hypotension arises from low cerebrospinal fluid pressure, typically resulting from an unseen leak [2,4]. These leaks occur in vulnerable regions around the dura mater and nerve root sheaths, often due to minor traumas such as falls, strenuous exercise, or forceful coughing, which can cause tears in the dura or arachnoid [2]. The source of a spontaneous cerebrospinal fluid leak may be either cranial or spinal, and the precise mechanism is still unclear [5]. Several etiologies have been postulated, and most evidences showed that leaks are predominantly due to mechanical tears, cerebrospinal fluid venous fistula, and leaking nerve root sleeves [6]. Schievink proposed four types of spontaneous spinal cerebrospinal fluid leaks [5]. Type 1 leaks result from degenerative disc disease, which leads to a mechanical tear occurring in the ventral dura. Type 2 leaks stem from lateral dural tears and are less common. Type 3 leaks occur due to dural defects that lead to cerebrospinal fluid venous fistula formation. Type 4 leaks involve distal nerve root sleeve leaks that do not form fistulas into the venous system but instead spread into the adjacent facial planes [6]. Moreover, individuals with connective tissue disorders, such as Marfan syndrome, are more susceptible to cerebrospinal fluid leaks. It is believed that structural vulnerabilities in the thecal sac increase the likelihood of meningeal tears following minor traumas or strenuous movements in these individuals [5]. Our patient did not exhibit any signs of a connective tissue disorder. On further investigation, when the patient was already in the recovery phase and undergoing rehabilitation, he disclosed that he had regularly used a handheld neck massager to alleviate neck stiffness while at work. Considering that a cerebrospinal fluid leak can also occur due to spinal manipulation, it is likely that our patient experienced a meningeal tear from the chronic mechanical stress caused by the neck massager.

Subdural hematoma commonly occurs due to bleeding from surface cerebral vessels following trauma. Substantial trauma is frequently necessary to cause the rupture of the bridging veins that traverse the narrow gap between the arachnoid membrane and the dura mater [1]. The exact cause of subdural hematoma in patients with spontaneous intracranial hypotension is uncertain; nevertheless, various studies have suggested several mechanisms. One theory is that the downward displacement of the brain resulting from low cerebrospinal fluid pressure might lead to tears in the bridging veins of the dural border cell layer, causing them to rupture [5]. Another possibility is that as subdural cerebrospinal fluid collections expand the subdural space over time, the bridging veins could stretch and potentially rupture in certain instances [5]. The signs of a subdural hematoma often develop gradually and may include headaches, altered consciousness, problems with walking or balance, cognitive impairment or memory problems, changes in behavior, and weakness in movement [7]. In this case, the patient had dizziness, drowsiness, and disorientation.

Cranial MRI is the imaging of choice to depict intracranial manifestations, which typically include pachymeningeal enhancement, brain sagging, small ventricles, bilateral subdural, and obliteration of cisterns [4,5,8]. Other specific quantitative parameters include a decreased interpeduncular angle [9] and decreased pontomesencephalic angle [8]. In our patient, non-contrast cranial MRI showed bilateral subdural hemorrhage, with a poorly visualized pontomesencephalic angle and interpeduncular angle.

In cases of spontaneous intracranial hypotension, the cerebrospinal fluid leak typically occurs in the cervical or thoracic region [5]. A whole spine MRI CISS protocol with contrast was performed on our patient, revealing a longitudinal spinal anterior epidural cerebrospinal fluid leak extending from spinal level C2 to T1. The precise location of the spinal cerebrospinal fluid leak is usually determined through CT myelography or radionuclide cisternography [6]. However, radionuclide cisternography of our patient did not exhibit definite scintigraphic evidence of extradural cerebrospinal fluid leak in both dynamic and delayed imaging. A cervical spine MRI CISS protocol with contrast was performed to monitor the extradural cerebrospinal fluid leak status, which showed stable longitudinal spinal anterior epidural cerebrospinal fluid leak from spinal level C2 to C7. The medical team concluded that since the cervical spine MRI CISS protocol with contrast showed stable cerebrospinal fluid leak findings, with gradual progressive improvement in the patient’s mental status, the cerebrospinal fluid leak might have spontaneously resolved or closed.

The initial approach to treating cerebrospinal fluid leaks usually involves conservative measures [10,11]. For some patients, such as in this case, recovery may be achieved through intravenous fluid hydration and maintaining the Trendelenburg position [2,10,11]. In this case, our patient underwent a specific regimen of Trendelenburg position and alternate flat position on the bed. This involved one and a half hours each day lying flat on the back with a 30-degree incline, with feet elevated above the head, alternated with two and a half hours of lying flat on the bed, with the head raised at a 30-degree angle to assist with enteral feeding and prevent aspiration. This cycle was repeated six times daily. On the fifth day of this regimen, the patient showed initial responsiveness to vigorous stimulation and demonstrated gradual improvement, culminating in spontaneous wakefulness by the 11th day. This treatment regimen operates on the principle that in patients with cerebrospinal fluid leakage, decreased craniospinal cerebrospinal fluid volume leads to a decrease in intracranial pressure (ICP) [2]. When transitioning from a lying down position to an upright position, the already diminished cerebrospinal fluid volume is further displaced to the spinal compartment [2]. This can lead to brain herniation due to pathologically low ICP, contributing to the observed decrease in sensorium in our patient. The Trendelenburg position is employed to counteract this phenomenon by elevating ICP through increased mean arterial pressure and reduced cerebral venous drainage [2]. The goal is to enhance the patient's sensorium by adjusting these physiological parameters. Continuing the regimen for a total of 14 days, the medical team observed positive progress. A subsequent cervical spine MRI with contrast revealed a stable longitudinal spinal anterior epidural cerebrospinal fluid leak, indicating potential resolution of the leakage.

## Conclusions

This case highlights the importance of a high index of suspicion and clinical history in the management of bilateral subdural hematoma without initial clear etiology. The Trendelenburg position demonstrates a crucial role in the management of intracranial hypotension by effectively increasing ICP. This positioning strategy offers a promising approach in ameliorating symptoms associated with cerebrospinal fluid leakage induced intracranial hypotension, thus potentially preventing complications such as brain herniation and improving patient outcomes. Further studies are warranted to elucidate optimal positioning protocols and long-term efficacy in clinical practice.
